# Meteorological Register

**Published:** 1834-01-01

**Authors:** 


### METEOROLOGICAL REGISTER,

FROM SEPTEMBER 1 TO NOVEMBER 30,

By Messrs. HARRIS and Co., Mathematical Instrument Makers, 50, High Holborn.

| | Thermometer. | | Barometer. | | De Luc's Hygrometer. | | Winds. | | Atmospheric Variation. |
|---|---|---|---|---|---|---|---|---|---|
| | *max.* | *min.* | *max.* | *min.* | *max.* | *min.* | | | |
| Sept. 1 to 7 | 67 | 43 | 30.07 | 29.24 | 66 | 61 | ENE | WNW | fine, cloudy, rain |
| 14 | 66 | 47 | 29.91 | .54 | 75 | 63 | E | NW | cloudy, fine, showery |
| 21 | 64 | 47 | 30.01 | .42 | 76 | 65 | NW | SW | fine, fine, cloudy |
| 30 | 73 | 48 | .01 | .40 | 75 | 71 | SE | SW | fine, rain, fine |
| Oct. 1 to 7 | 61 | 47 | .05 | .82 | 75 | 72 | E | SSE | fine, foggy, cloudy |
| 14 | 61 | 45 | .01 | .27 | 78 | 63 | ESE | SW | fine, foggy, rain |
| 21 | 59 | 40 | 29.53 | 28.83 | 79 | 71 | WNW | W | fine, cloudy, rain |
| 31 | 63 | 48 | .79 | 29.18 | 81 | 69 | S | SE | fine, cloudy, foggy |
| Nov. 1 to 7 | 60 | 35 | 30.02 | .24 | 80 | 70 | W | NW | rain, fine, cloudy |
| 14 | 59 | 36 | .06 | .56 | 80 | 76 | SW | NW | foggy, fine, rain |
| 21 | 53 | 37 | .06 | .66 | 84 | 77 | SSW | W | foggy, cloudy, fine |
| 30 | 56 | 34 | 29.94 | 28.63 | 83 | 74 | WSW | WNW | fine, foggy, rain. |

The quantity of Rain fallen in September, 2 inches and 25-100ths.
— — — October, 1 inch — 29-100ths.
— — — November, 1 inch — 15-100ths.

